# Microstructure and Lamellae Phase of Raw Natural Rubber via Spontaneous Coagulation Assisted by Sugars

**DOI:** 10.3390/polym13244306

**Published:** 2021-12-09

**Authors:** Wanna Bai, Jie Guan, Huan Liu, Shihong Cheng, Fuchun Zhao, Shuangquan Liao

**Affiliations:** School of Materials Science and Engineering, Hainan University, Haikou 570228, China; 18085204210001@hainanu.edu.cn (W.B.); guan_jie1223@163.com (J.G.); 19085204210030@hainanu.edu.cn (H.L.); biggercsh@hainanu.edu.cn (S.C.); shqliao@263.net (S.L.)

**Keywords:** natural rubber, spontaneous coagulation, sugars, crystallization, lamellae phase

## Abstract

Natural rubber (NR) as a renewable biopolymer is often produced by acid coagulation of fresh natural latex collected from *Hevea brasiliensis*. However, this traditional process is facing a huge economic and environmental challenge. Compared with the acid coagulation, spontaneous or microorganism coagulation is an ecofriendly way to obtain NR with excellent performance. To clarify the influence of different sugars on NR quality, several sugars were used to assist the coagulation process. Influence of different sugars on microstructure and cold crystallization were examined by ^1^H NMR, DSC, etc. The results indicated that sugars exhibit different biological activity on terminal components of fresh field latex and can influence the resultant molecular structure and basic properties. Brown sugar exhibits higher metabolic activity and is inclined to decompose the protein and phospholipids crosslinking compared with other sugars. The larger molecular weight of sugar molecule is beneficial for the formation of the stable *α* lamellae phase and higher overall degree of crystallization.

## 1. Introduction

Natural rubber (NR), an important renewable biopolymer material which is often produced via coagulation of the fresh natural latex collected from the tree *Hevea brasiliensis*, has been used in many applications for its outstanding properties [1]. Compared to its synthetic analogues, NR has relative excellent comprehensive properties such as extraordinary mechanical strength, superior tear strength, high crack growth resistance, excellent abrasion resistance, good biodegradability, low heat buildup and small hysteresis loss after several times of deformation [2,3,4,5,6,7,8].

Nowadays, technological processing of fresh natural latex coagulated by some types of acid is popular in the Chinese tropical area. However, this traditional process is facing the huge challenge of adapting to the rising severe environmental demands since the process will cost a large amount of ammonia and acid, which makes the traditional process neither economical nor eco-friendly. Additional treatment is needed to avoid pollution of the acidic effluent [9,10]. Moreover, in acid coagulation, the acid content in the coagulated rubber may be disadvantageous for some properties of the corresponding raw material rubber, for example the obvious tendency to scorch.

In recent years, the spontaneous or biological coagulation to obtain raw material natural rubber has attracted increasing attention due to not only their eco-friendly and economical nature, but also the excellent performance of NR, e.g., good mechanical properties, high cure rate, thermal stability, good dynamic mechanical properties [11,12,13,14]. Besides the protein, lipids, and minerals, sugars are one of the main non-rubber components. Previous studies have found that sugars play an important role in microbial metabolism and contribute to the basic characteristics and physical properties of natural rubber [1,3,15]. For example, white sugar is used to replace the acid used to coagulate the fresh latex and it is found that white sugar can promote reproduction of microorganisms in latex and produce abundant kinds of organic acids to coagulate the latex particles. The experimental results demonstrated that glucose not only affects the viscosity and the discoloration of NR but also have a strong effect on crosslink density, as well as tensile and dynamic properties of NR vulcanizates [3]. The Maillard reaction of glucose, fructose and ribose in natural rubber latex systems was investigated, and it was found that glucose and fructose can decrease the Mooney viscosity of DPNR and IR [16,17]. It was also found that fructose displays appreciable antioxidant activities [18]. Some carbohydrates could be involved in the mechanical properties of NR [19]. In addition, it is early reported that fructose and glucose have different activity in their utilization in respiration in natural latex system [20]. In our previous work, we also found that glucose will vary the content and exist state of molecular terminals and metal ions in raw natural rubber matrix and reconstruct the molecular network in raw NR via two main related action of glucose: metabolism and chelation [21]. It is obvious that sugars in fresh natural latex play an important role in the coagulation process and the quality of natural rubber due to their different ability of prompting the biochemical reaction occurred in the microorganism’s system. Based on the structure and purity difference of sugars, different kinds of sugars may exert a different influence on the microstructure and basic properties when they are used in the spontaneous coagulation of the natural rubber latex.

Moreover, in recent research of natural rubber, crystallization has attracted a considerable attention worldwide from both academic and industrial points of view due to its significant role in many mechanical performances and in some practical applications such as sealing [22,23,24,25,26]. According to the previous investigations, crystallization behavior of natural rubber is principally influenced by the fundamental chain structure or the component factors such as high cis-1, 4 isoprene unit content [27,28], branching structure [24], crosslinking [25], gel or 3D network [29], long-chain fatty acid ester content [30], and so on.

To our best knowledge, few studies have been focused on comparing the effect of sugars on the microstructure and crystallization behavior of NR. Information on the difference of the properties of raw natural rubber prepared by different kinds of commercial sugars in an assisted spontaneous coagulation process is not available in the literature so far. Hence, we studied the microstructure parameters and the cold crystallization behavior of raw NR with a view not only towards further insight of influence of sugars on basic properties but also the regulation and control of natural rubber quality. In order to clarify the difference of biological conversion and the influence of sugars on NR quality, several type of commercial sugars were used to assist the coagulation process of fresh field latex. Based on the difference of molecular degree of polymerization and purity of sugars, glucose, white sugar, brown sugar, trehalose, and cassava starch were used in this work. Metabolic activity of those sugars on terminal components, branching degree of natural rubber molecules, Mooney viscosity, rheological property were examined and the difference of cold crystallization behavior were also investigated.

## 2. Materials and Methods

### 2.1. Materials and Reagent

Fresh natural Latex was collected from Xida Farm located in Chengmai County, Hainan Province, China. Toluene (A.R.), glucose (A.R.) was purchased from Guangzhou chemical reagent factory, Guangzhou, China. Trehalose (99%) was manufactured by Hayashbara Co., Ltd., Okayama, Japan. White sugar (food-grade) and brown sugar (food-grade) were purchased from local supermarket. Cassava starch (food-grade) was from Wuxi Shenglunte international trade co. Ltd., Wuxi, China.

### 2.2. Samples Preparation

Raw NR was prepared via a spontaneous coagulation process assisted by the sugars. The typical process is as follows: First, the dry rubber content of the fresh natural rubber latex collected from the tree *Hevea brasiliensis* was determined by Microwave set (DH925A, Beijing Dahua Radio Instrument Factory, Beijing, China) according to the method provided in the instrument manual. Then, a quantitative volume of fresh natural latex containing 150 g dry rubber was diluted with deionized water to 20 wt% and then added 1 wt% aqueous sugar substance with slow stirring for 30 min. As for cassava starch, it needs to be gelatinized at 90 °C before being added into the latex. The above mixture was eventually adjusted to the 18 wt% of the dry rubber content to coagulate spontaneously in the stainless steel square plate with a glass cover at the room temperature until the wet gum formed completely. The coagulation and maturation were carried out at room temperature for around 60 h under typical tropical circumstances. Afterwards, the wet gum was washed, creped out, dried in an electro-thermostatic blast oven (DHG-9145A, Shanghai Yiheng Scientific Instrument Co., Ltd., Shanghai, China) for about 72 h to obtain the raw material natural rubber. The control sample was coagulated by acetic acid according to the traditional acid coagulation procedure.

### 2.3. Characterization

Molecular weight of NR samples were determined by viscometric method. The intrinsic viscosity, Huggins’ constant and Kramer’s constant were measured with a single bulb Ubbelohde viscometer (diameter 0.54 mm) at 30 ± 0.1 °C in toluene. All of the solvents and rubber solutions were filtered through a Bacteria funnel (30~50 micrometer) before measurement. The measurement was repeated until three consecutive readings differed by ±0.2 s. The intrinsic viscosity ([η]), Huggins’ constant ($k^{\prime }$) and Kramer’s constant (β) were calculated using the below Equations (1) and (2), respectively.

(1) $$\frac{\eta_{sp}}{c}=\left[\eta \right]+k^{\prime }{\left[\eta \right]}^{2}c$$

(2) $$\frac{ln\eta_{r}}{c}=\left[\eta \right]-\beta {\left[\eta \right]}^{2}c$$

where $\frac{\eta_{sp}}{c}$, $\frac{ln\eta_{r}}{c}$, [η], c, $k^{\prime }$ and β represent the reduced viscosity, logarithmic viscosity number, intrinsic viscosity, concentration expressed as g/dL, Huggins’ constant, and Kramer’s constant, respectively.

The ^1^H NMR measurement was carried out on Bruker AV 400 MHz instrument in CDCl_3_ at 25 °C. NR samples were swelled for 2 weeks in an NMR tube before conducting tests. About 1.2 mL g of 2 mg NR solution was used for ^1^H Spectroscopy analysis. The spectra scan number for ^1^H NMR measurements was 16.

Mooney viscosity (ML _1+4_) of natural rubber sample was performed using MV-2000 Mooney viscometer (Alpha Technologies, Wilmington, DE, USA). The sample is preheated at 100 ± 1 °C for 1 min before starting the motor and further continuously measured for 4 min with small size rotor. The measurement was repeated 3 times for each sample.

The measurement of rheological property was conducted on Rubber Processing Analyzer (RPA 2000, Alpha Technologies, Wilmington, DE, USA) at 100 °C, with the strain of 14% and sweeping frequency range of 0~30 Hz. The cylindrical test pieces (45 mm diameter and 4 mm thick) were prepared with a volumetric sample cutter after the homogenization of raw material rubber with a two-roll mill.

The raw natural rubber were first compression molded at 100 °C for 10 min under the pressure of 200 Kg/cm^2^. The mold was then cooled down to room temperature under pressure over 12 min. The resulting thin NR sheets (2 mm thick) were then die cut with type C dumbbell die. Determination of green strength was carried out using a Gotech AI-3000 model based on GB/T 528-92. The testing crosshead speed of 500 mm/min was applied with load cell of 100 N.

Crystallization of sample were measured by differential scanning calorimeter (DSC). About 10 mg natural rubber sample was sealed in standard Aluminum crucible to remove the thermal history by heating at 90 °C for 5 min and quickly cooled to crystallization temperature (T_c_ = −25 °C) for 8 h. After the end of crystallization, the sample was cooled to −40 °C and heated to 90 °C at the rate of 10 °C/min. According to the previous work, the degree of crystallization (*X_c_*) can be calculated by the following Equation (3).

(3) $$X_{c}=\frac{\Delta H_{f}}{\left(1-y\right)\Delta H_{f}^{{}^{\circ}}}$$

where *y* is the weight ratio of filler, ($\Delta H_{f}$) is the measured melting enthalpy. The melting enthalpy of perfect orthorhombic natural rubber ($\Delta H_{f}^{{}^{\circ}}$) equals 67.3 J/g [25,31].

## 3. Results and Discussion

### 3.1. Influence of Different Sugars on Terminal Components

**Figure 1:**
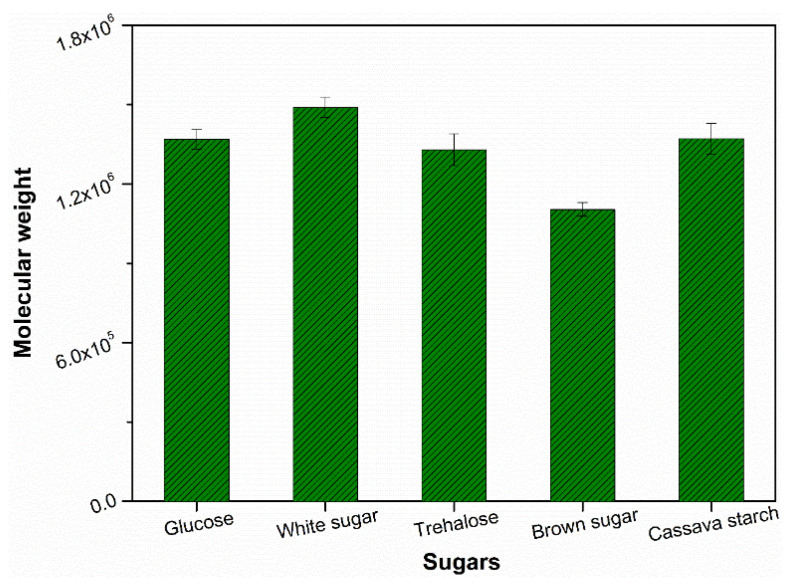
Effect of sugars used for coagulation on the molecular weight of resulting natural rubber.

It is well-known that factual molecular size of NR is related to the branching extent between single molecular chains. Our previous results demonstrated glucose exerted an obvious effect to adjust the number of ω-terminal and α-terminal of natural rubber molecular chains via the metabolism of microorganisms [21]. In order to compare the influence of different sugars on number of terminal components which can be indirectly reflected by the molecular chain size, the molecular weight of the raw natural rubber samples from auxiliary coagulation by different type of sugars was examined (shown in Figure 1). It can be seen that molecular weight of raw natural rubber with auxiliary coagulation by different sugars ranges from 1.10 × 10^6^ to 1.49 × 10^6^. As a control sample, the measured molecular weight of raw natural rubber coagulated by acid is 1.22 × 10^6^ which is only higher than that of sample from coagulation using brown sugar. Among those sugars used in the experiment, brown sugar resulted in the lowest molecular weight of raw natural rubber while white sugar got the highest molecular weight of raw natural rubber. Other sugars including glucose, trehalose and cassava starch resulted in medium molecular weight. This may be attributed to molecular activity of different sugars due to their different molecular structure and purity. Although brown sugar and white sugar both belong to the disaccharide, they have different activity to promote biological action of microorganism in the latex system due to their nuanced component and chemical structure. Brown sugar usually contains some other nutritious constituents such as vitamins and trace mineral which are beneficial to biological action of microorganism. Thereafter, brown sugar is inclined to decompose the protein and phospholipids crosslinking and obtain the lower molecular weight. In other words, the effect of sugars on the resulted molecular weight can be ascribed to their different ability of decomposing the initial crosslinking points between the natural rubber molecular chains through prompting the metabolism of microorganism.

**Figure 2:**
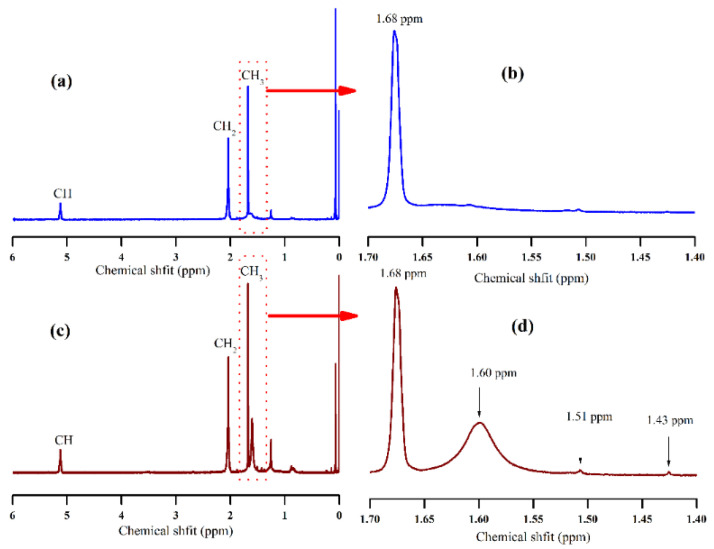
^1^H NMR spectra for NR samples coagulated by (**a**) acetic acid and (**c**) brown sugar. Spectra (**b**,**d**) are the partial enlargement of (**a**,**c**) at chemical shift range from 1.70 to 1.40, respectively.

To further confirm the ability of sugar to decompose the protein and phospholipids, ^1^H NMR measurement was carried out on the NR sample coagulated by traditional acid and sugars, respectively. Since the above molecular weight data indicated that the brown sugar has a higher ability of this kind, brown sugar was chosen as the typical representation of sugars to compare the traditional acid. ^1^H NMR spectra for NR samples from brown sugar and from acetic acid (see Figure 2). Three major signals at 1.68 ppm, 2.04 ppm and 5.12 ppm appearing on spectra (a) and (c) are assigned to the methyl proton (CH_3_), methylene proton (CH_2_) and methine proton (CH) of cis-1, 4-isoprene unit of NR molecular chain, respectively. Compared with spectrum (b), spectrum (d) appeared additional signals at 1.60 ppm, 1.51 ppm, 1.43 ppm which indicated that brown sugar has the ability to decompose the proteins and phospholipids. This was consistent with the previous reference [32] which testified that signals between 1.4 ppm and 1.8 ppm were attributed to the decomposing the branching points associated with the linked fatty acids due to transesterification.

**Figure 3:**
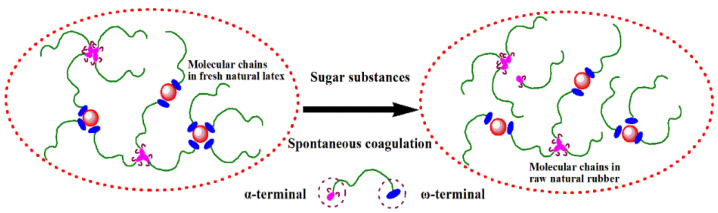
Schematic illustration of the possible decomposition of crosslinking points between the terminals of the natural rubber molecular chains.

The possible decomposition of crosslinking points between the terminals of the natural rubber molecular chains was shown in Figure 3. In the specific natural latex system, sugars and other terminal components together make up the recipe of nutritious medium for prompt the microorganism’s metabolism. Specific microorganisms in latex have different metabolism activity due to the different molecular structure and impurity of sugars. If some kind of sugars can dramatically increase metabolism in which the terminal component may be consumed or be converted into small molecules at the same time, the number of branching point originated from crosslinking or associating of terminal components such as lipid or protein accordingly decreased and resulted lower molecular weight.

### 3.2. Branching Degree of Natural Rubber Molecules

**Figure 4:**
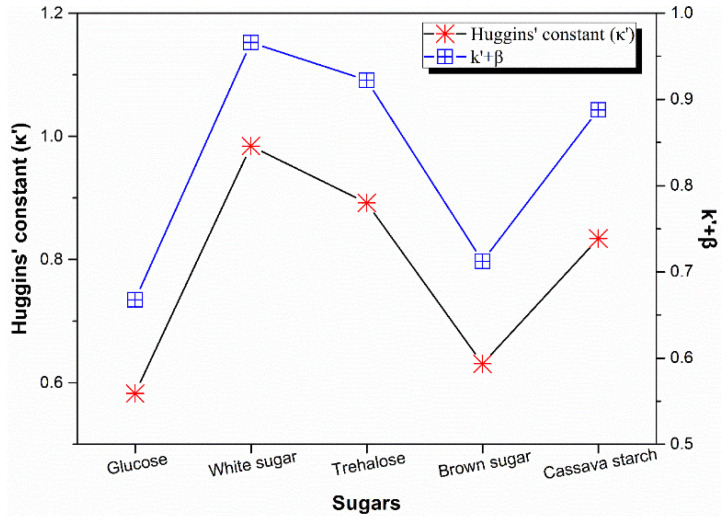
Effect of sugars on *k*′ and *k*′ + *β*.

Figure 4 showed the effect of different sugars on the Huggins’ constant (*k*′) and the sum value of Huggins’ constant and Kramer’s constant (*β*). *k*′ and *β* can be used to describe the branching degree of natural rubber molecules [1]. *k*′ is the qualitative index of long chain branching, which goes up with the increment of the number of branching while it is independent of molecular weight and molecular weight distribution. If *k*′ ranges from 0.45 to 0.65, it shows that macromolecules are branched; if *k*′ is about 0.3, the macromolecules are linear [33]. In regard to the good solution of linear polymers, the sum of *k*′ and *β* equals 0.5, However, the value of the sum of *k*′ and *β* has apparent deviation from 0.5.

From the Figure 4, it can be seen clearly that coagulation using brown sugar and glucose led to the lower *k*′ value and the sum of *k*′ and *β* is very close to the 0.5, which indicating that the corresponding NR sample from auxiliary coagulation by brown sugar and glucose may have a lower branching degree and the rubber chains are close to linear molecules. While white sugar gained a higher *k*′ value and the sum of *k*′ and *β* deviated from 0.5. Therefore, the sample of natural rubber solidified by white sugar had a higher branching degree. Trehalose and cassava starch got a middle *k*′ value. In addition, the sum of *k*′ and *β* had a similar tendency to *k*′ with respect to the all sugars used in the work, which indicated the parameter of *k*′ agreed well with the parameter of the sum of *k*′ and *β* in assessing of the branching degree of the natural rubber molecular chains.

### 3.3. Mooney Viscosity

**Figure 5:**
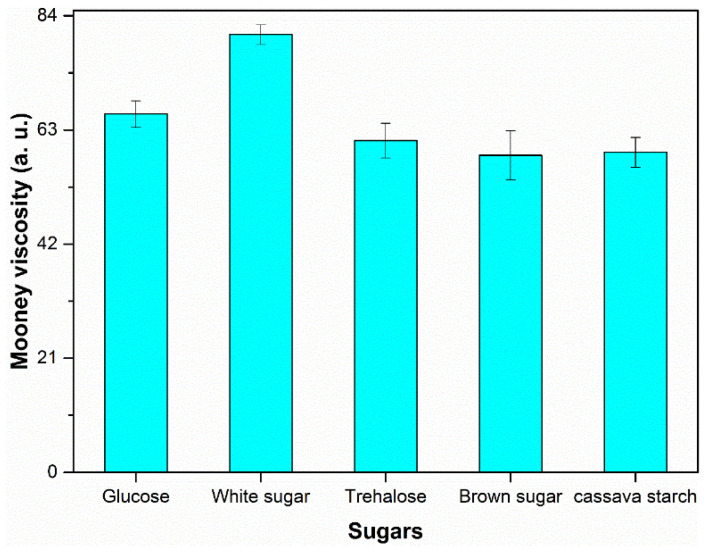
Effect of different sugars on Mooney viscosity of NR samples.

Mooney viscosity which indicates the flow properties of NR is an important assessment parameter for assessing the raw material natural rubber quality and processability [34]. The Mooney viscosity results of samples coagulated by different sugars were shown in Figure 5. The natural rubber sample resulted from coagulation using white sugar showed the highest Mooney value. It can be found that the influence of sugar type on the tendency of Mooney viscosity changes and on molecular weight in this experiment were very similar (see Figure 1). Since Mooney viscosity of the corresponding raw natural rubber sample mainly depends on the molecular weight, this can further confirm that metabolic activity of different sugars on terminal components.

### 3.4. Rheological Behavior

**Figure 6:**
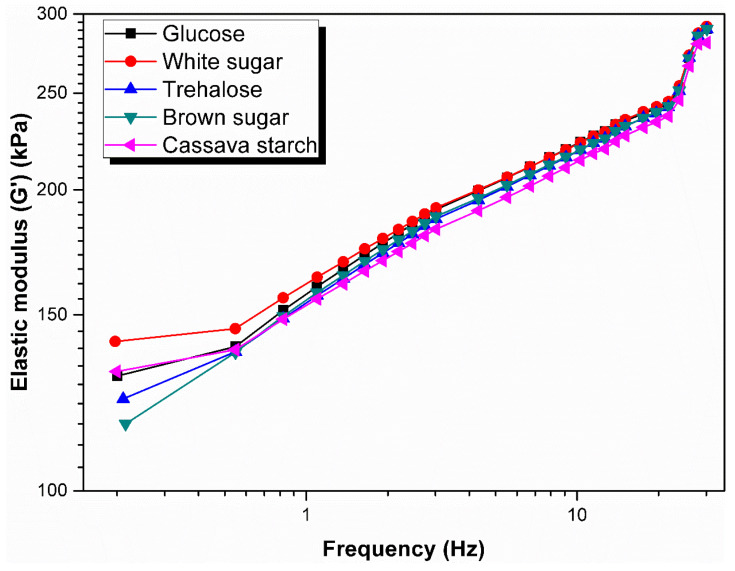
Elastic modulus as a function of frequency of raw natural rubber resulted from coagulation with different sugars.

Rubber Processing Analyzer (RPA) was widely used to determine the rheological properties of rubber melts [35,36]. The change in elastic modulus with frequency may indicate more insight into the polymer microstructure [37]. Figure 6 shows the elastic modulus (G’) as a function of frequency for raw natural rubber resulted from coagulation with different sugars on a double logarithmic plot. The sweeping was carried at strain of 14% and 100 °C and the frequency ranged from 0 to 30 Hz. With the increment of the oscillation frequency, the G’ for all raw natural rubber rose. However, the larger change in the G’ was more prominent at lower frequency range, and it became minimal at higher frequencies, and the curves were inclined to gather in one point. According to Shao [38], higher molecular weight melts contribute the higher G’ due to their larger entanglement density. Thus, the higher G’ of the rubber sample from coagulation with white sugar indicated that it had a higher molecular weight while the lower G’ of the rubber sample from coagulation with brown sugar indicated a lower molecular weight, the intermediate values of G’ of samples coagulated with other three kind of sugar used in the experiment are in an agreement with the previous molecular weight results (see molecular weight results in Section 3.1).

**Figure 7:**
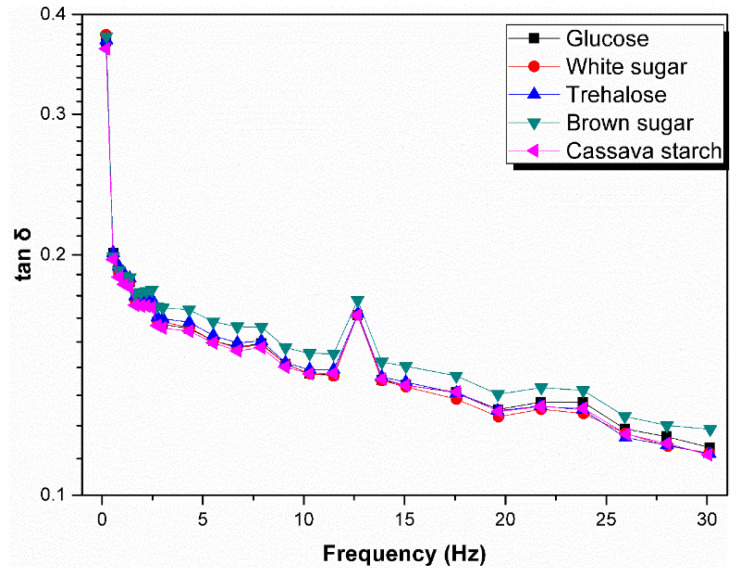
Tanδ as function of frequency of raw natural rubber resulted from different sugars.

Moreover, the loss factor (tanδ) which is defined as the ratio of viscous modulus to elastic modulus reflects the recovery characteristics of the melts after deformation and a larger tanδ value indicates a more liquid-like character [38]. Figure 7 shows the tanδ as function of frequency of raw natural rubber resulted from coagulation using different sugars. It can be seen that the values of tanδ decreased significantly at lower frequency region and slowly at higher frequency region. In addition, among the sugars used in the experiment, brown sugar renders the rubber sample with higher tanδ, which means that the corresponding rubber sample has a relative higher plasticity and a smaller deformation in the range of the processing oscillation frequency and demonstrates a good processing performance.

**Figure 8:**
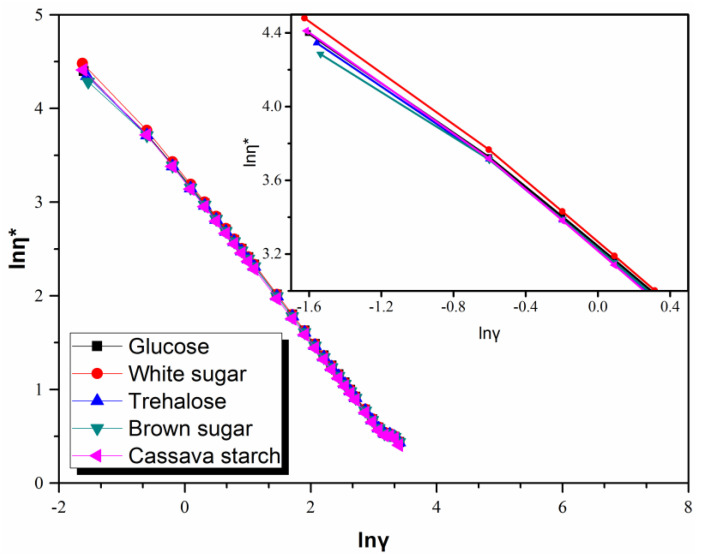
Relationship between lnη* and lnγ of raw natural rubber resulted from coagulation using different sugars.

Figure 8 shows the relationship between logarithmic value of complex viscosity (η*) and lnγ at 373 K for raw rubber samples from coagulation using different sugars. It can be seen that all rubber samples showed pseudoplasticity and the corresponding shear thinning behavior. As shown in the inset of Figure 8, rubber sample from coagulation using white sugar also exhibited a slightly higher complex viscosity within the low-frequency region in comparison with that from coagulation using brown sugar, which can be attributed to the influence of sugar on the molecular weight and branching structure of natural rubber molecular chains. Coagulation with brown sugar results in a relatively lower molecular weight and degree of branching abovementioned that means a nethermore entanglement density and weaker interaction between molecular chains, thus the rubber sample from coagulation with brown sugar exhibits a lower complex viscosity at lower frequency.

### 3.5. Green Strength

**Figure 9:**
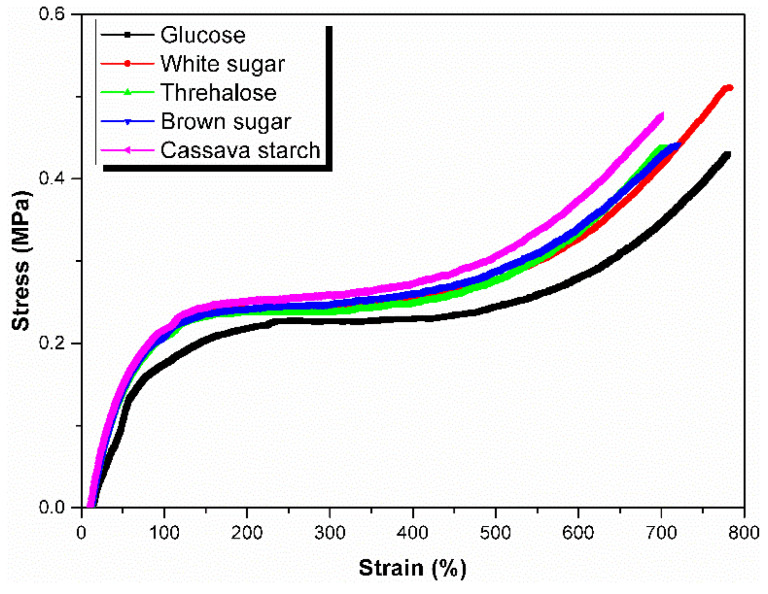
Stress–strains curves for unvulcanized rubbers from coagulation using different sugars.

Sugars exist different biological action with non-rubber constituent and have different decomposition effect on the branching points of the natural rubber molecular chains which will consequently affect the stress-strain behaviors of the unvulcanized rubber that are important to certain processing operations in the rubber industry [33]. The green strength of an elastomer is its resistance to deformation and fracture before vulcanization [39]. To determine the green strength, the stress-strain behavior of unvulcanized NR prepared from coagulation using different sugars was investigated, and the stress-strain curves are shown in Figure 9. It can be seen that coagulation with white sugar and cassava starch resulted in the higher green strength values, which were basically consistent with the tendency of previous data of molecular weight (see Section 3.1) and the Huggins’ constant (see Section 3.2). In other words, both the type of sugars that facilitated keeping the structure of long-chain branching and further less lowering the molecular weight of natural rubber molecular chains will lead to the higher green strength. The detail data of tensile properties of the unvulcanized rubber sample from coagulation using different sugars substance are listed in Table 1. It is clear that the natural rubber sample from coagulation using white sugar exhibited the highest elongation at break, whereas the sample from Cassava starch coagulation using gave the least value. The stiffness and hardness of the rubber sample can be demonstrated by 100 modulus and 300% modulus indirectly. It is noted that 100 modulus and 300% modulus as well as tensile strength presented a similar trend. The rubber samples from coagulation using white sugar and cassava starch had the higher corresponding value of 100 modulus and 300% modulus. Higher green strength, 100 modulus and 300% modulus may be attributed to the higher branching degree. The higher branching degree means more crosslinking points between the two terminals of natural rubber molecular chains. Thus, the higher branching degree plays a role in restraining the mobility of the molecular chains under strain conditions and present a higher corresponding value.

### 3.6. Crystallization Behavior

**Figure 10:**
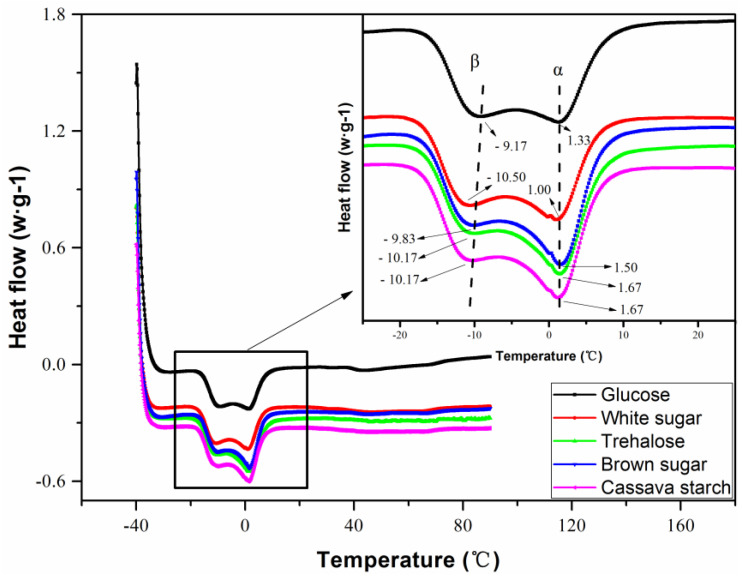
DSC melting curves of NR sample coagulated by different sugars.

For the cold crystallized natural rubber c natural rubber, there are always two different peaks detected in the DSC curves. According to Philips [40], the lower melting peaks could be attributed to thermodynamically less stable *β* lamellae and the higher melting peak could be produced by thermodynamically more stable *α* lamellae. Chenal [25] showed that the lower temperature peak was responsible for the phase of *β* lamellae, and the higher temperature peak for the phase of *α* lamellae. Therefore, the influence of sugar type on the formation of *α* lamellae and *β* lamellae was determined by DSC (see Figure 10). From the inset of Figure 10, it can be clearly seen that using different sugar resulted in different peak positions or peak areas of *α* lamellae and *β* lamellae, respectively. It is commonly accepted that peak position of melting peak is related to lamellae thickness and crystal perfection [25,41]. The *β* peak shifted slightly toward lower temperature with increasing molecular weight of sugar roughly, while *α* peak shifted toward higher temperature very slightly. However, the shift of *α* peak was much smaller than that of the *β* peak. This indicated that *α* lamellae phase was thicker and or/more perfect when lower molecular weight of sugar used in the spontaneous coagulation of fresh natural latex. The sequence of *β* peak position towards the higher temperature was glucose, brown sugar, trehalose and Cassava starch, and white sugar.

**Figure 11:**
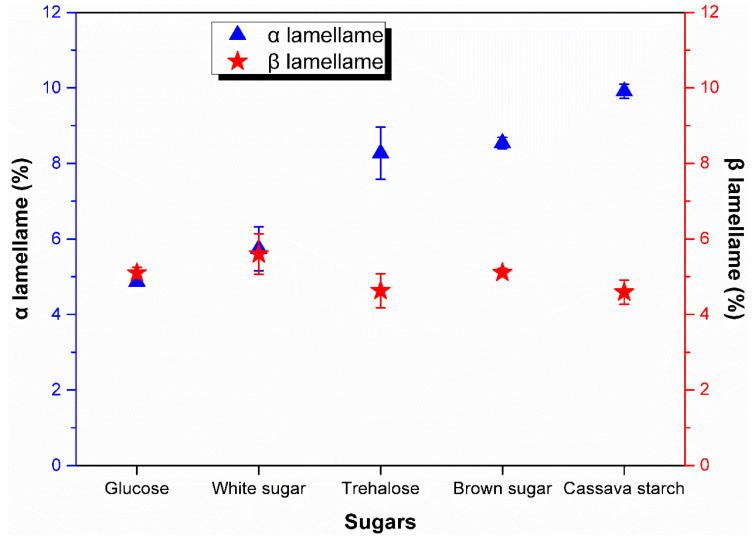
Effect of sugar substance on the fraction of *α* lamellae and *β* lamellae.

Based on the soft-ware built in the Mettler-Toledo DSC system, the melting enthalpy and the crystallinity of both *α* and *β* lamellae were calculated (see Figure 11). Figure 11 gave out the influence of sugar substance on fraction of *α* lamellae and *β* lamellae. The fraction of *α* lamellae and *β* lamellae of control sample was also determined and was 7.37% and 6.03%, respectively, which indicates that the fraction of *β* lamellae of rubber samples from coagulation with these sugars was lower than that from control sample. It is obvious that glucose as a monosaccharide resulted in the higher content of less stable *β* lamellae than stable *α* lamellae among all sugars used in the experiment. Except for the sample from the assisted coagulation by glucose, the fraction of *α* lamellae was higher than that of *β* lamellae in the samples from the assisted coagulation by other disaccharides and polysaccharide. It is noteworthy that the ratio of *α* lamellae to *β* lamellae was increasing with the molecular weight of sugar. That is to say, the ratio of *α* lamellae to *β* lamellae of rubber samples would be higher from coagulation with monosaccharide to disaccharide and then polysaccharide. Additionally, polysaccharide was in favor of formation stable lamellae *α* and higher total degree of crystallization.

**Figure 12:**
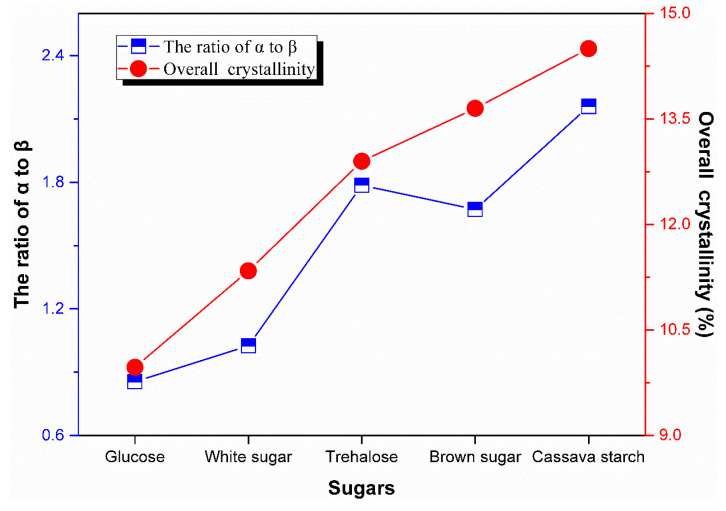
Effect of sugars on the ratio of *α* to *β* and overall crystallinity.

Figure 12 shows the ratio of *α* lamellae to *β* lamellae and the overall crystallinity of rubber sample coagulated with different kinds of sugars. The fraction of *α* lamellae and *β* lamellae of control sample was also determined and was 7.37% and 6.03%, respectively, which indicated that the fraction of *β* lamellae of rubber samples from coagulation with these sugars was lower than that from control sample. It is obvious that the overall crystallinity in the natural rubber sample increased with the increase of molecular weight of sugars used in the experiment. Moreover, the effect of sugars on the ratio of *α* to *β* in crystallographic phase was roughly similar to the overall crystallinity. Among the three kinds of disaccharides, trehalose resulted in higher ratio of *α* to *β*.

## 4. Conclusions

A green way to obtain raw material natural rubber was completed via assisted coagulation of natural latex by sugar substance. In this process, microorganisms exhibit different biological activity with different sugars and the given terminal components of original fresh field latex as nutrition and further influence the resultant molecular structure and basic properties including molecular weight, branching degree, Mooney viscosity, rheological performance and green strength. Cold crystallization behavior as an important performance for the natural rubber is also influenced by different sugars. The saccharide molecules of larger molecular weight are beneficial to the formation of the stable *α* lamellae phase and higher overall degree of crystallization. The results may provide an alternative way for the conversion of field coagula into suitable forms for long-term storage and marketing.

## Figures and Tables

**Table 1:** Mechanical properties of unvulcanized natural rubber samples obtained by coagulation with different sugars.

Sugars	Tensile Strength (MPa)	Elongation at Break (%)	100% Modulus (MPa)	300% Modulus (MPa)
Glucose	0.43 ± 0.03	780.3 ± 33.0	0.17 ± 0.01	0.23 ± 0.01
White sugar	0.51 ± 0.08	780.6 ± 60.0	0.21 ± 0.03	0.24 ± 0.03
Trehalose	0.44 ± 0.08	705.0 ± 76.4	0.21 ± 0.01	0.24 ± 0.00
Brown sugar	0.44 ± 0.01	719.5 ± 9.3	0.21 ± 0.01	0.25 ± 0.01
Cassava starch	0.48 ± 0.02	699.8 ± 33.6	0.22 ± 0.01	0.26 ± 0.02

## Data Availability

The data presented in this study are available on request from the corresponding author.
